# Safety of Rifampicin at High Dose for Difficult-to-Treat Tuberculosis: Protocol for RIAlta Phase 2b/c Trial

**DOI:** 10.3390/pharmaceutics15010009

**Published:** 2022-12-20

**Authors:** Juan Espinosa-Pereiro, Samiksha Ghimire, Marieke G. G. Sturkenboom, Jan-Willem C. Alffenaar, Margarida Tavares, Sarita Aguirre, Arturo Battaglia, Gladys Molinas, Teresa Tórtola, Onno W. Akkerman, Adrian Sanchez-Montalva, Cecile Magis-Escurra

**Affiliations:** 1 International Health Unit Vall d’Hebron-Drassanes, Infectious Diseases Department, Vall d’Hebron University Hospital, Department of Medicine Universitat Autónoma de Barcelona, Programa de Salut Internacional del Institut Catalá de Salut (PROSICS), 08035 Barcelona, Spain; juan.espinosa@vallhebron.cat; 2 Centro de Investigación Biomédica en Red de Enfermedades Infecciosas (CIBERINFEC), Instituto de Salud Carlos III, 28029 Madrid, Spain; 3 Department Clinical Pharmacy and Pharmacology, University Medical Center Groningen, University of Groningen, 9700 RB Groningen, The Netherlands; s.ghimire@umcg.nl (S.G.); m.g.g.sturkenboom@umcg.nl (M.G.G.S.); johannes.alffenaar@sydney.edu.au (J.-W.C.A.); 4 School of Pharmacy, Faculty of Medicine and Health, The University of Sydney, Sydney, NSW 2006, Australia; 5 Westmead Hospital, Sydney, NSW 2145, Australia; 6 Sydney Institute for Infectious Diseases, The University of Sydney, Sydney, NSW 2006, Australia; 7 Infectious Diseases Service, Centro Hospitalar de São João, 4200-319 Porto, Portugal; margarida.tavares@chsj.min-saude.pt; 8 National Program for Tuberculosis, Ministry of Health, Asunción 1430, Paraguay; sarita.aguirre79@gmail.com; 9 Instituto Nacional de Enfermedades Respiratorias y Ambientales, Asunción 1430, Paraguay; arturombc@hotmail.com (A.B.); gladys_molinasleon@hotmail.com (G.M.); 10 Microbiology Department, Vall d’Hebron University Hospital, 08035 Barcelona, Spain; mariateresa.tortola@vallhebron.cat; 11 TB Center Beatrixoord, Haren, University Medical Center Groningen, University of Groningen, 9751 ND Groningen, The Netherlands; o.w.akkerman@umcg.nl; 12 Department of Pulmonary Diseases and Tuberculosis, University Medical Center Groningen, University of Groningen, 9700 RB Groningen, The Netherlands; 13 Grupo de Estudio de Infecciones por Micobacterias, Sociedad Española de Enfermedades Infecciosas y Microbiología Clínica (GEIM-SEIMC), 28003 Madrid, Spain; 14 Radboud University Medical Centre, Department of Respiratory Diseases-TB Expert Center Dekkerswald, 6561 KE Nijmegen, The Netherlands; cecile.magis-escurra@radboudumc.nl

**Keywords:** tuberculosis, rifampicin, clinical trial, vulnerable population

## Abstract

Previous clinical trials for drug-susceptible tuberculosis (DS-TB) have shown that first-line treatment with doses of rifampicin up to 40 mg/kg are safe and increase the early treatment response for young adults with pulmonary tuberculosis. This may lead to a shorter treatment duration for those persons with TB and a good baseline prognosis, or increased treatment success for vulnerable subgroups (age > 60, diabetes, malnutrition, HIV, hepatitis B or hepatitis C coinfection, TB meningitis, stable chronic liver diseases). Here, we describe the design of a phase 2b/c clinical study under the hypothesis that rifampicin at 35 mg/kg is as safe for these vulnerable groups as for the participants included in previous clinical trials. RIAlta is an interventional, open-label, multicenter, prospective clinical study with matched historical controls comparing the standard DS-TB treatment (isoniazid, pyrazinamide, and ethambutol) with rifampicin at 35 mg/kg (HR^35^ZE group) vs. rifampicin at 10 mg/kg (historical HR^10^ZE group). The primary outcome is the incidence of grade ≥ 3 Adverse Events or Severe Adverse Events. A total of 134 participants will be prospectively included, and compared with historical matched controls with at least a 1:1 proportion. This will provide a power of 80% to detect non-inferiority with a margin of 8%. This study will provide important information for subgroups of patients that are more vulnerable to TB bad outcomes and/or treatment toxicity. Despite limitations such as non-randomized design and the use of historical controls, the results of this trial may inform the design of future more inclusive clinical trials, and improve the management of tuberculosis in subgroups of patients for whom scientific evidence is still scarce. Trial registration: EudraCT 2020-003146-36, NCT04768231.

## 1. Introduction

Current standard treatment for drug susceptible tuberculosis (DS-TB) was designed 40 years ago and has remained unchanged except for a 4-month regimen using high-dose rifapentine and moxifloxacin following the results of a recent clinical trial [1]. Rifampicin is still the backbone drug in the treatment of DS-TB because of its potent bactericidal and sterilizing activity. The currently used once daily 10 mg/kg dosing of rifampicin was selected in the 1970s and was based on pharmacokinetics, toxicity, and cost concerns [2]. Under clinical trial conditions and using per protocol analysis, the current standard regimen achieves a 95% efficacy [3]. However, several pharmacological, bacterial, and host factors may negatively affect treatment outcomes in daily practice.

Some groups of people are more vulnerable to TB because of risk factors (e.g., advanced age, immunosuppression, diabetes, or liver disease) that increase their risk of active disease and poor treatment outcomes [4]. For example, treatment success rate was 86% globally, but only 77% for people living with HIV [5]. Drug-induced liver injury is the best-known adverse reaction associated with rifampicin, isoniazid, and pyrazinamide. In addition, rifampicin typically causes gastrointestinal symptoms and hypersensitivity reactions (pruritus, rash, flu-like syndrome, hemolytic anemia). Although for clinicians these groups are also considered more vulnerable to adverse reactions, different studies show contradictory results. For example, in a retrospective study including 1149 patients, age > 60, diabetes, and hepatitis B virus (HBV) infection were not associated with an increased risk of adverse reactions [6].

Optimal drug regimen, dose, and treatment duration are still not well established, as these vulnerable groups were underrepresented in previous clinical trials as these were conducted in mid-to-high TB burden countries. However, the population in these studies included young participants with pulmonary TB and no comorbidities except 20% of participants with HIV coinfection (data from a systematic review by the European South American TB Research Collaborative Network (EUSAT-RCS, manuscript in preparation). A rifampicin dose ranging trial in DS-TB patients by Boeree and colleagues reported that doses (up to 50 mg/kg) were safe and tolerated, and further improved the extended early bactericidal activity in TB patients [7,8]. The optimized doses achieved up to 10-fold higher exposure in plasma (AUC0-24) and a higher early bactericidal activity measured by a decline in the colony-forming units count in sputum culture [7,9,10]. A meta-analysis of 13 studies from 1979 to 2021, including adults with TB meningitis and pulmonary TB receiving doses up to 35 mg/kg, did not show an increase in the incidence of Severe Adverse Events (SAE), with a pooled Incidence Risk Ratio of 1.00 (95% confidence interval 0.82–1.23) [11]. Therefore, increased doses might have the potential to shorten DS-TB treatment. Pharmacokinetic studies show that a low proportion of patients reach target exposures with the current standard doses of rifampicin. For example, in a clinical trial in Indonesia, only 48% of the participants receiving standard dose rifampicin reached the 8 mg/L threshold for maximum plasma concentrations (Cmax) deemed to be effective [12]. In addition, adults with TB and other comorbidities such as HIV and diabetes may have lower exposure to rifampicin [13]. In the absence of actual AUCs, clinicians often use the concentration 2 h after drug intake as a predictor of rifampicin exposure [14].

The RIAlta study aims to evaluate the safety and efficacy of rifampicin at 35 mg/kg daily for the intensive phase of the first line TB treatment in vulnerable adults with TB, usually excluded from clinical trials due to age and/or comorbidities. This is a single arm prospective study using matched historical controls. This article summarizes the trial protocol and the rationale behind key aspects of its design.

## 2. Methods

### 2.1. Study Design

This is an interventional, open-label, multi-center, historically matched controlled prospective clinical study of high dose rifampicin (35 mg/kg, HR^35^ZE group) versus standard-dose (10 mg/kg/day, historical HR^10^ZE group) in a vulnerable population. Clinical trials with rifampicin are typically open label as the drug causes orange discoloration of the urine and other body fluids that makes it difficult to maintain blinding for participants and investigators [15,16,17]. Table 1 shows a comparative of key design aspects in different phase 2 studies using high-dose rifampicin in the last 15 years.

### 2.2. Objectives and Endpoints

The study primary objective is to evaluate the safety of high-dose rifampicin (35 mg/kg/d) as part of a standard first line regimen using standard doses of isoniazid (5 mg/kg), pyrazinamide (25 mg/kg), and ethambutol (15–25 mg/kg) for 8 weeks in adult subjects with pulmonary or extrapulmonary DS-TB belonging to difficult to treat subgroups. The secondary objectives are to evaluate the tolerability, efficacy, bactericidal activity, pharmacokinetic/pharmacodynamics, and cost effectiveness of high dose rifampicin, among others. 

The primary safety endpoint is the proportion of participants and historical controls with one or more grade 3 or higher adverse events (AE) according to the CTCAE v5 or SAE at 8-weeks after treatment with high-dose rifampicin onset. Table 2 below describes in detail the analysis population and primary safety outcomes. The secondary tolerability endpoint includes the proportion of participants with any AE. The secondary efficacy endpoint includes the proportion of participants and historical controls who have sputum culture conversion at 8 weeks after treatment onset or a proportion of participants with clinical improvement according to the treating physician (if follow-up images are available for extra-pulmonary TB) but without a follow up sample at 8 weeks after treatment onset.

Most TB phase 2 trials complete their intervention at week 8 or earlier and then participant care is transferred to a public institution within the National TB programs, so the information about end of treatment and post-treatment outcomes is scarce [10,17,18]. In the RIAlta trial, after the intervention and the primary safety objective evaluation, an extended follow-up period will be offered to participants, in line with the phase 2b/c design suggested by Phillips et al. [19]. This will provide information about end-of-treatment outcomes and relapse incidence in the prospective group.

### 2.3. Study Setting

The European South American TB Research Collaborative Network (EUSAT-RCS) is a consortium supported by the European Commission (Marie Sklodowska-Curie grant number 823890). The study will be carried out at four different TB treatment centers across Europe and one in South America. Vall d’Hebron Institute of Research (VHIR, Barcelona, Spain) is the sponsor of the study, and Hospital Universitario Vall d’Hebron (Barcelona, Spain) is the coordinating center and serves as the primary contact point for this trial. Other participating centers include: Radboud University Medical Center, Nijmegen, the Netherlands; University Medical Center Groningen, Groningen, the Netherlands; Centro Hospitalario Universitário de São João, Porto, Portugal; and Instituto Nacional de Enfermedades Respiratorias y del Ambiente (INERAM), Asuncion, Paraguay.

According to WHO 2020 data, TB incidence per 100,000 population is low in The Netherlands, Spain, and Portugal, whereas it is still high in Paraguay [5]. Diabetes prevalence is estimated to be 15% among people with TB, in contrast with 9.3% globally in 2019 [20]. Median age is between 42 and 46 years in Europe, whereas in high burden countries, the population is some 20 years younger on average (26 years in Paraguay, 27 in South Africa). Both South America and the European region have relatively low incidence rates of HIV-TB coinfection (3 in 100,000) [5]. In a UK prospective study, 18.1% of the patients with active TB had markers for hepatitis B or C infection were significantly higher than the 0.3% prevalence for the general population in the same period [21]. However, despite the need to study the possible increase in incidence, the potential drug interactions, and the risk of drug-induced liver injury with TB treatment, these coinfections are exclusion criteria for many TB trials (Table 1).

### 2.4. Study Population and Eligibility 

Adult participants ≥18 years with confirmed or probable pulmonary or extrapulmonary TB will be eligible to participate in the study. Additionally, trial candidates must be older than 60 years or have one of the following: diabetes, HIV, HBV or HCV coinfection, malnutrition (body mass index below 18.5 kg/m^2^), or any other chronic but stable liver disease. 

Adult participants with *M. tuberculosis* that is rifampicin-resistant by Xpert MTB/RIF or by drug susceptibility testing; with a Barthel index < 40 or life expectancy of less than 2 months regardless of anti-TB treatment; with signs of hepatotoxicity characterized by AST or ALT > 5x upper limit of normal, total bilirubin > 3x upper limit of normal, Child–Pugh grade C cirrhosis or acute decompensation at enrollment, or any grade 3–4 hepatobiliary alteration; who were previously treated with first-line anti-TB drugs or quinolones for at least 14 days or current treatment for more than 7 days; with solid organ or bone marrow transplantation; with active neoplasm requiring chemotherapy or immunotherapy treatment; or with previous severe pulmonary disease other than TB according to the investigator’s judgment will be excluded from the study. Participants with aischemic heart disease OR severe arrhythmia within 6 months OR subjects with atrial fibrillation and indication of oral anticoagulant therapy when transitioning to low-molecular weight heparin is not feasible will also be excluded from the study. Lastly, pregnant or breastfeeding women and participants not suitable to be included according to the investigator will not be enrolled for the study.

This is one the key differential aspects of RIAlta’s design. Previous trials have included people living with HIV, but few participants with diabetes, age over 60, or chronic liver disease were included [11,22].

### 2.5. Recruitment

Participants will be prospectively included and compared with historical controls from the same sites. Participants meeting all the inclusion and not any of the exclusion criteria will be offered to start on the HR^35^ZE arm. After 8 weeks, participants will be managed according to the national TB guidelines.

The selected retrospective participants will provide a comparison for the primary safety outcome, and to the secondary efficacy outcome of culture conversion at 8 weeks using MGIT media. 

We adhered to all Pockock’s principles for historical controls except for the use of information from participants that were previously randomized in clinical trials [23]. To avoid the selection bias that may arise because of this limitation, we aim to include all adult patients fulfilling the inclusion criteria treated with HR^10^ZE in the participating sites between January 2017 and December 2019 (to avoid the influence of the 2020–2021 SARS-CoV-2 pandemic in TB diagnoses and follow-up). Thus, the records of all patients from that period with pulmonary and extrapulmonary rifampicin susceptible tuberculosis will be reviewed. We will capture the severe or grade 3 adverse events and week 8 microbiological status from those who are ≥60 years, or ≥18 years and have a diagnosis of diabetes, malnutrition, HIV, HBV or HCV coinfection, other stable chronic liver diseases, or TB meningitis, and have no pharmacological immunosuppression. We intend to control the retrospective cohort to match the prospective one for age, sex, cavitation in lung TB, and severity in TB meningitis. In case not enough matched controls are not found, we will extend the search with patients until the calculated sample size in the control arm is achieved. The historical cohort will include participants from only Spain, Portugal, and Paraguay and not likely from the Netherlands, as optimized doses of rifampicin have been used in both Dutch sites for more than a decade in the same participant subgroups as included in our study.

### 2.6. Intervention

**Figure 1:**
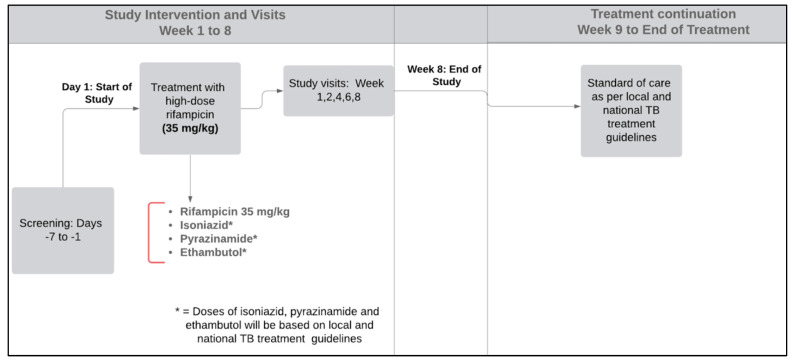
Study flow diagram.

The intervention in this study is a once daily high-dose of rifampicin at 35 mg/kg. A dose-finding study conducted in South Africa found that 40 mg/kg/day was the maximum tolerated dose in young participants without significant comorbidities [8]. Participants should start anti-TB treatment as soon as possible after signing written informed consent. This protocol allows a window of up to 7 days to complete screening tests (whether TB treatment was initiated or not; see study design diagram: Figure 1). Pyridoxine (vitamin B6) supplementation will be allowed as per local guidelines recommendations. The investigation team will carefully assess other concomitant drugs. When interactions are detected, dose adjustments or substitutions by an alternative drug will be at the discretion of the investigators.

### 2.7. Rifampicin Dosing Strategies

Study participants will receive high dose rifampicin 35 mg/kg for 8 weeks (maximum once daily dose of 3150 mg) by combining fixed-dose combination (FDC) tablets with rifampicin loose capsules (Table 3). We have adapted the WHO guidelines weight band dosing scheme for a more accurate rifampicin dosing. The dose for each band was calculated for the medium weight in each band (45 mg × 35 mg = 1575 mg, rounding 1500 mg), and for those with >80 kg, the dose is calculated for 90 kg (90 mg × 35 mg = 3150 mg).

One limitation is the scarcity of the 600 mg presentation for rifampicin, which would reduce the pill burden for the participants in this trial. For instance, as optimized doses of rifampicin come closer to daily practice, public health decision makers should advocate to increase the availability of rifampicin in high dose presentations to reduce the number of pills taken by patients.

To achieve a similar drug exposure to oral rifampicin at 35 mg/kg/day, the dose for intravenous administration should be 26 mg/kg/day [13].

### 2.8. Assessment of Study Outcomes, Analysis Groups, and Duration of Follow-Up

The primary safety hypothesis is that the incidence of adverse events in the high dose rifampicin (35 mg/kg) supplemented with isoniazid, pyrazinamide, and ethambutol [HR^35^ZE] during the first 8 weeks of treatment will not exceed that of controls on a standard HRZE regimen (rifampicin 10 mg/kg) [HR^10^ZE]. The HR^35^ZE regimen will be considered unsafe if in a non-inferiority analysis, the upper limit of the 95% confidence interval of the difference of proportions in grade ≥ 3 AE (according to CTCAE scale version 5) between the experimental and the historical control arm exceeds 8%. The most common adverse events related to therapy with higher rifampicin doses were vomiting, headache, hyperuricemia, pain in the extremities, and pruritus. No significant differences according to rifampicin dose (from 10 to 35 mg/kg) were found in a recent systematic review and in a retrospective study collecting the real-life experience from a Dutch center [24,25].

To assess the AE and ensure a homogeneous evaluation of their grade, severity, expectedness, and treatment relatedness, we will administer a standardized training for the investigators from all different sites. In addition, both for the prospective and the retrospective cohorts, grade 3 or higher adverse events and SAE will be reviewed by the trial’s Steering Committee for relatedness classification. When no consensus is reached, the Data and Safety Monitoring Board formed by experts independent from the trial will make the final decision.

Tolerability will be measured as any adverse event (grade 1 to 4), treatment dropout rate, and any dose reduction. For efficacy outcome, the hypothesis is that in DS-TB participants (pulmonary disease), the proportion of participants experiencing successful treatment outcomes (culture and smear negative) at the end of the intensive phase of treatment on HR^35^ZE will be non-inferior to that of controls on HR^10^ZE. The HR^35^ZE regimen will be considered ineffective if the lower limit of the 95% CI of the proportion of participants with a negative sputum culture (sputum sterilization), or unable to produce sputum if they have a previous negative culture at week 8, is more than 10% lower than that of historical controls treated with HR^10^ZE. Other exploratory outcomes will be reported descriptively. 

After the trial intervention ends at week 8, participants will be transferred to a standard of care as per national guidelines. We will extend the follow-up duration up to one year after the completion of the treatment to capture relapse-free cures. 

We will use different subsets of the study population for the primary safety, tolerability, and efficacy objectives. Table 2 shows a detailed analysis plan as described in the protocol. 

### 2.9. Sample Size Assumptions and Justification

For the sample size calculation, a non-inferiority design is assumed and the upper limit of the 95% confidence interval (CI) of the difference of proportions in grade 3 or higher adverse events at 8 percentage points has been set. We assume that 5% of patients will not be assessable. Alpha risk is set in 0.1 and power in 0.8. Based on the previous literature and our previous experience, expected standard treatment grade ≥ 3 AE are 5–10% (mean 8%) among experimental arms and standard treatment arms. Considering this, the number of participants per arm has been calculated to be 134 participants. Because of the limited recruitment capacity for this trial, we could not target specific numbers of participants for each sub-group of risk factors. 

Based on a feasibility survey performed at the planning stage, the target enrollment rates per country were: Paraguay 6.25 participants per month; Spain, Portugal, and the Netherlands 1.6 participants per month.

### 2.10. Main Outcomes Analysis

The primary safety outcome will be analyzed as the proportion of participants in the HR^35^ZE and historical HR^10^ZE groups that received at least one dose of treatment with an unfavorable outcome after 8 weeks of experimental treatment start. According to the clinical and laboratory information, participants will be classified as having an unfavorable outcome if they suffered one or more grade ≥ 3 AE or SAE that are possibly, probably, or definitely related to rifampicin OR they did not complete their treatment because of other reasons OR were lost-to follow-up. High doses of rifampicin will be considered non-inferior to standard doses if the upper boundary of the 95% CI of the proportion of participants with an unfavorable outcome is less than 8% that of the point estimate in the historical standard dose group.

Efficacy will be assessed as the proportion of participants with pulmonary TB in the HR^35^ZE and the historical HR^10^ZE groups that have sputum culture conversion in liquid media at week 8. The unfavorable efficacy outcome will be defined as having a positive sputum culture at week 8.

### 2.11. Secondary Objectives

Secondary objectives can be grouped into 4 categories. The analyses will be descriptive as these data will not be available from most of the historical HR^10^ZE controls.

#### 2.11.1. Microbiological Evaluation

We will collect sputum samples from the experimental treatment pulmonary TB participants at week 1, 2, 4, 6, and 8, and compare the baseline bacterial load with the bacterial load at each of these time points as estimated by the time to positivity in days, as compared to that at baseline [9]. Time to positivity (TTP) is a good correlate of bacterial load and this information is readily available from the MGIT system. In addition, we should be able to estimate CFU count using a formula previously published [26]. In addition, we will compare the changes in TTP with a baseline and follow-up breath signal of the AeoNose™ device, an electronic nose that analyzes volatile organic compounds (VOC) in exhaled air. Pilot studies show that the VOC signal decreases during treatment; hence, it has potential as a biomarker to estimate bacterial load [27].

#### 2.11.2. Pharmacokinetics

Drug exposure is a better predictor of treatment success than the total dose. There is extensive pharmacokinetic data on rifampicin at doses up to 50 mg/kg/day, but the target population in RIAlta was not represented. To capture rifampicin exposure in steady state, we will collect PK samples at week 4 after experimental treatment starts following a simplified sampling strategy (2, 4, and 6 h) to predict exposure (AUC0-24) [28].

#### 2.11.3. Pharmacogenetics

The genes involved in rifampicin metabolism and hepatotoxicity, whose expression is modified by rifampicin itself, are thought to explain the wide inter-individual variations observed in rifampicin exposure and development of toxicities among certain individuals. The RIF gene is mutated at three sites: CYP2C9*2, CYP2C9*13, and CYP2C19*2, which might have significance in drug-induced liver injury. The study by Su et al. found that the CYP2C9*2 genotype was significantly associated with drug-induced liver injury [29]. In Europeans and admixed Americans, CYP3A4*22 is the most common allele (minor allele frequency, MAF 5% and 2.6%, respectively) with *3 and *2 contributing to the genetic variability in the former study. In contrast, CYP3A4*15 (MAF 2.5%) and CYP3A4*18 (MAF 1.9%) constitute the only common CYP3A4 alleles in Africans and East Asians [30]. A subset of participants will be offered to participate in a genetic analysis searching for variants in the genes *SLCO1B1*, *ABCB1*, *UGT1A,* or *PXR*. We will correlate these results with the exposure obtained from the PK sub-study as well as with the clinical and microbiological outcomes [31]. 

#### 2.11.4. Health Economics and Quality of Life

We will assess the costs (direct and indirect), the incidence of catastrophic costs (those that are ≥20% of the household’s annual income), and the changes in quality of life associated with the use of high dose rifampicin in this trial population. The EUSAT-RCS consortium has developed a survey tool to capture the costs for TB patients and their caregivers based on that described by the WHO [32,33].

### 2.12. Ethical Approvals

The trial protocol has been registered in EudraCT and Clinicaltrials (EudraCT 2020-003146-36, NCT04768231 respectively), and has already been approved by the Medical Ethics Committees from Vall d’Hebrón Hospital in Barcelona, Spain, and Radboudumc in Nijmegen for The Netherlands (approval number: NL75346.091.20). Additional ethics approval will be acquired by each participant country. 

### 2.13. Sharing of Trial Findings

Trial results regarding its main safety and efficacy outcomes, and all secondary outcomes will be summarized in the final trial report and submitted to regulatory authorities and ethics committees as required in each participating country. In addition, the results will be made public through peer-reviewed journals, and the generated datasets will be made available according to the European Commission requirements and data protection regulations.

### 2.14. Participant and Public Engagement

No structured community engagement was followed during the design of this trial. We will engage with participants, TB patient associations, and the community to discuss the results of this study and contextualize them and ensure a relevant impact. Furthermore, these results will inform the design of future projects, in which a continuous dialogue with the TB research community will be of utmost importance.

### 2.15. Strengths and Weaknesses of the Design

The population included in the RIAlta trial includes people from groups typically excluded from clinical trials, and will thus outline the applicability of optimized doses of rifampicin, with exploratory data on late clinical efficacy endpoints (relapse-free survival). The simultaneous collection of pharmacokinetic, microbiologic, and a breath signature will help provide a complete perspective on the intervention.

However, the non-randomized design and the use of historical controls make the RIAlta trial especially prone to bias. Although preventive measures have been taken, the results should be considered as informative to future trial designs rather than general practice changing.

## 3. Discussion

RIAlta is a multinational, open label, non-randomized phase 2b/c trial that aims to bridge an important knowledge gap on the safety of optimized rifampicin doses in vulnerable TB patients that are more prone to suboptimal treatment outcomes and drug toxicities because of age and comorbidities. The trial will provide useful information on the efficacy and pharmacokinetics of 35 mg/kg rifampicin dosing and explore possible correlations between occurring adverse events and the rifampicin genetic polymorphisms involved in metabolism and hepatotoxicity. It will evaluate the usefulness of exhaled VOCs as biomarkers of early treatment response. Finally, the study will evaluate the social and economic circumstances of the participants.

There are no validated surrogates for early treatment response for extra-pulmonary TB. Efficacy studies at 8 weeks based on sputum culture conversion does not include this patient population. The cure at the end of treatment is also difficult to confirm in this group because of the need of invasive samples that are not indicated as the procedure might pose extra/unnecessary risks to patients’ lives. As a result, most of the trials exclude persons with extrapulmonary TB and the safety and efficacy information are extrapolated from pulmonary TB studies. It is well known that the pharmacokinetics of antimicrobials may vary according to the site of infection [13]. To address this, our study will obtain information about pulmonary and extrapulmonary TB in parallel. The correlation between intermediate data about sputum culture conversion and change in sputum bacterial load as estimated by the TTP for pulmonary TB participants and change in VOC in all participants will enable us to explore if the breath signature changes could be used as surrogate markers for extra-pulmonary TB. This could open the path for non-invasive biomarker validation and future inclusion of people with extrapulmonary TB in regulatory trials.

Therefore, the population included in this study belong to the other risk factors groups for failure and relapse that have been under-represented in previous clinical trials [4,22]. As a phase 2 study, the main aim of this clinical trial is to evaluate safety of rifampicin defined as the rate of participants suffering grade 3 or higher adverse events or severely adverse events. This information will be likely available from historical controls although it was not systematically collected. Grade 1–2 adverse events, which are considered tolerability problems, are not typically reported in clinical records, or the information about them is not complete, thus making it difficult to make comparisons. To address this, we have developed adverse events monitoring and management guidelines, which will be harmonized at all centers for the prospective part. Extensive training will be provided to the investigators at each site. 

It is important to acknowledge the main limitations related to this trial design. Because of the limited recruitment capacity within our consortium and budgetary constraints, we decided to use historical controls (rifampicin 10 mg/kg along with the same TB regimen) and assign all prospectively recruited participants to the 35 mg/kg dose. This results in a non-randomized design as all participants will be assigned to the experimental arm and compared with historical controls. Hence, another limitation is that information about tolerability is often incomplete or not reported in clinical records, and the endpoints in the different sub-studies are not performed in daily clinical practice. For similar reasons, the sample size is too small for a formal subgroup analysis. The results will inform a larger phase 3 trial with power calculations for relevant subgroups.

Having one PK sampling at week 4 will allow us to show the percentage of participants reaching the target exposure (AUC/MIC) when in a steady state after rifampicin induction of its own metabolism. In contrast, this approach will not provide information about exposure during the first week of treatment, when most of the bacterial killing occurs. Nevertheless, this knowledge will aid our understanding if optimized rifampicin dosing is more likely to meet target rifampicin exposure. 

Any attempt to improve TB treatment outcomes must also consider its impact on the socio-economic background of the people who suffer because of TB. This includes patients with active TB infection and extends to their families and communities. A treatment that is safe and effective, but not well tolerated or takes longer to treat has consequences not only on individuals and families but also on a society. For example, frail TB patients might be absent from the workforce for a longer time, threatening financial security of the whole family in informal economies. Furthermore, caregiving activities of a family member might be over-stretched to meet the needs of patients that affects both direct and indirect costs of TB. Hence, the global impact of a new treatment scaled to a programmatic level could be unforeseen inconveniences for the patients and their families beyond the biological aspects of health. 

In the past few decades, very few clinical trials on TB have been conducted in the European region because of low TB incidence (and hence low recruitment speed) and increased costs as compared to other regions. For those conducted, none of the trials have focused on the use of an optimized rifampicin dose in the targeted patient population [34]. European sites are still important for external validation and, importantly, to offer innovative treatment opportunities for people with TB. In addition, whereas implementing clinical trials in low-and middle-income countries has specific logistic challenges, working closely in both realities through collaborative consortiums such as ours, EUSAT-RCS, creates a unique milieu for capacity building. Via mutual exchange of personnel and expertise, researchers can develop the skills, knowledge, and competencies needed to plan, design, and execute clinical trials adapted to the local setting while adhering to the international ICH-GCP guidelines. In the long term, the implementation of this trial will help the establishment of a sustainable research network integrating EU and non-EU sites.

## 4. Conclusions

This paper provides a roadmap for using historical controls and setting up multi-center, multi-national pragmatic trials in real-life settings for diseases such as TB that take a long time to treat and follow up, and for which research resources are rather scarce. We believe that the results from the RIAlta study will inform future, larger trials and thus contribute to the generalization of the use of optimized doses of rifampicin in the real-world setting.

## 5. Trial Status

The trial is registered with the EudraCT number 2020-003146-36 and the NCT04768231. Site initiation visits and the recruitment of the first participants are expected to start in the last quarter of 2022 or first quarter of 2023. 

## Figures and Tables

**Table 1:** Comparison of recent phase 2 trials using high-dose rifampicin for tuberculosis.

Study	Trial ID	Design	Population	Key Exclusion Criteria	Arms	Max. Rifampicin (mg/kg/day)	Follow-Up (Weeks)
Ruslami 2013	NCT01158755	Phase 2b, open label, randomized	≥15 years old with clinically suspected TB meningitis	Body weight < 30 kg, liver disease	2	13	26
HR1	NCT01392911	Phase 2a, open label, sequential allocation	18–65 years old with confirmed pulmonary DS-TB	High alcohol consumption, diabetes	7	50	2
RIFATOX	ISRCTN55670677	Phase 2b, open label, randomized	18–65 years old with confirmed pulmonary DS-TB	High alcohol consumption, diabetes, HIV	3	20	16
HIGH RIF-2	NCT00760149	Phase 2b, double blind, randomized	18–65 years old with confirmed pulmonary DS-TB	Body weight < 50 kg, clinical hepatitis/cholestasis	3	20	12
MAMS-TB	NCT01785186	Phase 2b,c, open-label, randomized	≥18 years old with confirmed pulmonary DS-TB	High alcohol consumption, HIV infection with <200 CD4	5	35	48
HIRIF	NCT01408914	Phase 2b, double blind, randomized	18–60 years old with confirmed pulmonary DS-TB	Body weight < 30 kg, viral hepatitis	2	20	26
RifT	ISRCTN42218549	Phase 2, open label, randomized	≥18 years old with clinically suspected TB meningitis	Cirrhosis or clinical jaundice	3	35	24
RIFAVIRENZ	NCT01986543	Phase 2, open label, randomized	≥15 years old with confirmed pulmonary DS-TB	AIDS-defining infection, pharmacological immunosuppression	2	20	28
RIAlta	NCT04768231	phase 2b,c, open label, historical controls	≥60 or ≥18 years old (with HIV, HBV, HCV, DM), pulmonary and extrapulmonary TB	Decompensated chronic liver disease, oral anticoagulation	1	35	72

**Table 2:** Analysis populations and endpoints.

Assessment	Primary Safety	Efficacy
1. Per protocol population	Includes participants who completed assigned follow-up AND were at least 80% adherent to study treatment.	Includes those participants who complete assigned follow-up AND were at least 80% adherent to study treatment.
2. Intention to treat population	Includes all the participants that received at least one dose of anti-TB drugs.	Includes all the participants enrolled in the study.
3. Modified intention to treat population	Includes a subset of intention-to-treat population with exclusion of some subjects in a justified way (subjects having non-tuberculous mycobacteria, early withdrawal of consent, etc.).	Includes subset of intention-to-treat population with exclusion of some subjects in a justified way (participants having non-tuberculous mycobacteria, early withdrawal of consent, etc.).
4. Microbiological intention to treat population	Includes all the participants that received at least one dose of anti-TB drugs AND have culture confirmation of DS-TB.	Includes all the participants enrolled in the study AND have culture confirmation of DS-TB.
5. Microbiological per-protocol population	Includes those participants who complete assigned follow-up AND were at least 80% adherent to study treatment AND have culture confirmation of DS-TB.	Includes those participants who complete assigned follow-up AND were at least 80% adherent to study treatment AND have culture confirmation of DS-TB.

**Table 3:** Weight-band dosing of rifampicin.

High Dose Rifampicin (35 mg/kg)	Weight-Band Dosing					
	35–40 kg	41–50 kg	51–60 kg	61–70 kg	71–80 kg	≥80 kg
**Rifampicin tablets** (300 mg)	900 mg 3 tablets	900 mg 3 tablets	1500 mg 5 tablets	1500 mg 5 tablets	1800 mg 6 tablets	2400 mg 8 tablets
**Rifampicin tablets** (150 mg)	-	150 mg 1 tablet	-	150 mg 1 tablet	150 mg 1 tablet	-
**Fixed dose combination** (*150 mg rifampicin, 75 mg isoniazid, 400 mg pyrazinamide, and 275 mg ethambutol*)	450 mg 3 tablets	450 mg 3 tablets	450 mg 3 tablets	600 mg 4 tablets	750 mg 5 tablets	750 mg 5 tablets
**Total rifampicin dose**	1350 mg	1500 mg	1950 mg	2250 mg	2700 mg	3150 mg

## Data Availability

Not applicable.
